# Getting ahead of Alzheimer’s disease: early intervention with focused ultrasound

**DOI:** 10.3389/fnins.2023.1229683

**Published:** 2023-07-27

**Authors:** Rebecca L. Noel, Samantha L. Gorman, Alec J. Batts, Elisa E. Konofagou

**Affiliations:** ^1^Department of Biomedical Engineering, Columbia University, New York, NY, United States; ^2^Department of Radiology, Columbia University, New York, NY, United States

**Keywords:** focused ultrasound, Alzheimer’s disease, blood–brain barrier, spatial memory, long-term memory, anxiety, amyloid, tau

## Abstract

The amyloid-β (Aβ) hypothesis implicates Aβ protein accumulation in Alzheimer’s disease (AD) onset and progression. However, therapies targeting Aβ have proven insufficient in achieving disease reversal, prompting a shift to focus on early intervention and alternative therapeutic targets. Focused ultrasound (FUS) paired with systemically-introduced microbubbles (μB) is a non-invasive technique for targeted and transient blood–brain barrier opening (BBBO), which has demonstrated Aβ and tau reduction, as well as memory improvement in models of late-stage AD. However, similar to drug treatments for AD, this approach is not sufficient for complete reversal of advanced, symptomatic AD. Here we aim to determine whether early intervention with FUS-BBBO in asymptomatic AD could delay disease onset. Thus, the objective of this study is to measure the protective effects of FUS-BBBO on anxiety, memory and AD-associated protein levels in female and male triple transgenic (3xTg) AD mice treated at an early age and disease state. Here we show that early, repeated intervention with FUS-BBBO decreased anxiety-associated behaviors in the open field test by 463.02 and 37.42% in male and female cohorts, respectively. FUS-BBBO preserved female aptitude for learning in the active place avoidance paradigm, reducing the shock quadrant time by 30.03 and 31.01% in the final long-term and reversal learning trials, respectively. Finally, FUS-BBBO reduced hippocampal accumulation of Aβ40, Aβ42, and total tau in females by 12.54, 13.05, and 3.57%, respectively, and reduced total tau in males by 18.98%. This demonstration of both cognitive and pathological protection could offer a solution for carriers of AD-associated mutations as a safe, non-invasive technique to delay the onset of the cognitive and pathological effects of AD.

## 1. Introduction

Alzheimer’s disease (AD) is the most common form of dementia, accounting for over 55 million cases worldwide as of 2023.^1^ As an age-associated neurodegenerative disease, elderly people comprise the population most affected, however, 4–5% of AD cases can be classified as early-onset (EOAD), appearing in patients younger than 65 years old (Mendez, 2012). Symptomatically, AD impairs episodic memory, visuospatial functioning, communication, attention, cognition, and executive functioning (see text footnote 1) (Weller and Budson, 2018; Breijyeh and Karaman, 2020). The most pervasive and earliest symptoms of AD are deficits in memory encoding and recall (Soria Lopez et al., 2019, p. 13). In addition to these hallmark cognitive symptoms, AD is associated with neuropsychiatric symptoms such as apathy (49% prevalence), depression (42%), aggression (40%), anxiety (39%), and disordered sleep (39%) (Zhao et al., 2016). The detection of amyloid-β (Aβ) plaques *in vivo* using positron emission tomography (PET) or in cerebrospinal fluid (CSF) may contribute to a positive AD diagnosis (Marcus et al., 2014; Breijyeh and Karaman, 2020). Indicators of neuronal injury, such as morphological abnormalities or brain atrophy detected by magnetic resonance imaging (MRI) may also confirm AD diagnoses (Frisoni et al., 2010; Tondelli et al., 2012; Breijyeh and Karaman, 2020). The necessity of both cognitive and pathological metrics for AD diagnoses requires an analogous improvement of both for an effective, qualified AD therapeutic.

Focused ultrasound-induced blood–brain barrier opening (FUS-BBBO) is a non-invasive, targeted method for transiently opening the blood–brain barrier (BBB) for drug delivery or neuroimmune modulation (Choi et al., 2007; Ji et al., 2021; Hosseini et al., 2022; Kline-Schoder et al., 2022; Batts et al., 2023). FUS-BBBO involves the focusing of acoustic pressure waves inside the brain, which oscillate systemically introduced microbubbles (μB) to mechanically disrupt the endothelial cells lining cerebral vessels, causing a local, transient increase in BBB permeability (Hynynen et al., 2001; Sheikov et al., 2008).

The etiology of AD is under active investigation and debate (Karran et al., 2011; Breijyeh and Karaman, 2020). However, the pathophysiology of AD is characterized by Aβ plaque and tau neurofibrillary tangle (NFT) accumulation, a breakdown in BBB integrity, inflammation, synaptic dysfunction, and neurodegeneration. The amyloid-cascade hypothesis states that amyloid is the primary driver of AD progression. In AD, the transmembrane amyloid precursor protein (APP) is cleaved by β- and γ-secretase to form either Aβ40 or Aβ42 (Barage and Sonawane, 2015). Aβ accumulation begins in cerebral brain regions before spreading to the neocortex, allocortex, and brainstem (Hampel et al., 2021). Aβ40 is more prevalent and more innocuous by comparison to the more hydrophobic and subsequently more prone to aggregation, Aβ42 (Barage and Sonawane, 2015). The increase in Aβ accumulation in the brain likely results from variable transporter expression by endothelial cells and pericytes at the BBB (Dá Mesquita et al., 2016). The tau hypothesis provides an additional explanation for the progression of AD clinical symptoms centered on hyperphosphorylated tau. Tau is a microtubule-associated protein responsible for mediating microtubule stability (Wang and Mandelkow, 2016; Kametani and Hasegawa, 2018; Mondragón-Rodríguez et al., 2020). Tau phosphorylation is naturally regulated to mediate microtubule binding (Barage and Sonawane, 2015). However in AD, tau hyperphosphorylation causes inappropriate dissociation from microtubules, the formation of neuron-destabilizing NFTs, impaired axonal transport, and disrupted neuronal functioning (Mondragón-Rodríguez et al., 2020; Ju and Tam, 2021). NFTs spread from the entorhinal cortex, to the hippocampus and finally to cortical brain regions (Takeda, 2019).

It has been demonstrated that the permeability of the BBB and its susceptibility to disruption with FUS increase with age and AD in the hippocampi of humans and preclinical models, respectively (Montagne et al., 2015; Noel et al., 2023). In response to Aβ accumulation, microglia and astrocytes increase inflammation in the brain and BBB by releasing inflammatory cytokines and chemokines that can lead to oxidative stress, neurodegeneration, demyelination, synaptic damage, and apoptosis (Barage and Sonawane, 2015; Fakhoury, 2018; Leng and Edison, 2021). Interestingly, astrocytes and microglia may also respond favorably in AD pathological conditions by facilitating Aβ clearance (Fakhoury, 2018; Leng and Edison, 2021). Overall, the elevated activation and inflammatory state of the AD brain contributes to the degeneration and onset of cognitive symptoms in AD.

Genome-wide association studies performed on AD patients have implicated several risk genes in the development of EOAD. In particular, mutations in presenilin 1 and 2 (PSEN1 and PSEN2), as well as APP have been linked to elevated levels of secreted Aβ protein (Scheuner et al., 1996; Mendez, 2012; Barage and Sonawane, 2015; Ju and Tam, 2021). Additionally, the APOEε4 and APOEε2 alleles of the apolipoprotein E (APOE) gene, are especially interesting in AD (Karran et al., 2011; Mendez, 2012; Long and Holtzman, 2019; Ju and Tam, 2021). While inheriting the APOEε2 allele confers neuroprotection, inheriting a single copy of the APOEε4 allele increases the likelihood of developing late-onset AD by 300–400%, and increases the rate and quantity of Aβ deposition (Long and Holtzman, 2019). Due to the nature and early-intervention timeline of preventative therapies, carriers of AD-associated genetic mutations represent the target patient population for this kind of AD therapy (Barage and Sonawane, 2015).

The safety of transcranial FUS-BBBO has been well-established in multiple animal models and most recently, in human clinical trials (Baseri et al., 2010; Tung et al., 2011; McDannold et al., 2012; Samiotaki et al., 2012; Downs et al., 2015; Lipsman et al., 2018; Pouliopoulos et al., 2021; Batts et al., 2022). FUS-BBBO has been shown to increase drug delivery efficiency, promote neurogenesis, stimulate a neuroimmune response, reduce AD pathology, and improve cognition across multiple preclinical, therapeutic studies in late-stage AD models (Burgess et al., 2014; Scarcelli et al., 2014; Leinenga and Götz, 2015; Karakatsani et al., 2019; Ji et al., 2021; Leinenga et al., 2021; Hosseini et al., 2022). Outside of AD, FUS has been indicated for non-invasive tumor ablation and clinically approved for thalamotomy in essential tremor patients (Fishman, 2017; Mehkri et al., 2023). The ability to locally and transiently increase BBB permeability makes FUS-BBBO an important tool for treating various conditions in the brain, especially in combination with other therapeutic modalities such as targeted antibodies or endovascular chemotherapy (Leinenga et al., 2021; Patel et al., 2022).

In humans, several therapeutic attempts have been made centering on the Aβ hypothesis, attempting to either reduce Aβ production and accumulation or increase Aβ clearance (Long and Holtzman, 2019; Ju and Tam, 2021). The failure of several Aβ-targeting drugs and clinical trials to significantly improve clinical AD symptoms has raised concerns about this approach (Gauthier et al., 2016; Kwan et al., 2020; Yiannopoulou and Papageorgiou, 2020). Additionally, the post-mortem discovery of extensive amyloid in the limbic and association cortices of otherwise healthy adults has weakened the Aβ hypothesis (Haass and Selkoe, 2007). Other therapeutics targeting tau-related mechanisms of AD progression have been developed, which attempt to maintain the stability of microtubules, reduce tau aggregation and production, or increase the clearance of tau protein (Ju and Tam, 2021). These therapies range from vitamins, to small molecule drugs, to monoclonal antibodies that bind and label the target protein for engulfment and degradation (Long and Holtzman, 2019; Ju and Tam, 2021; Oumata et al., 2022). Unfortunately, many of these attempts have failed to substantially rescue cognition and disease progression, showing insufficient efficacy in Phase 3 clinical trials including patients with mild-to-moderate AD (Long and Holtzman, 2019).

A variety of prevention studies have been attempted in transgenic AD murine models varying from targeted antibodies or metal compounds to implementing specific exercise routines or diets (García-Mesa et al., 2011; Noristani et al., 2012; Giménez-Llort et al., 2013; Morse, 2022). These studies report various levels of pathological and cognitive improvement compared to untreated controls. However, many begin after the onset of pathology *in vivo*, making it difficult to discern if the intervention is truly preventing disease onset, or rather treating the disease symptoms.

The insufficiency of late-stage AD interventions and the promise of previous early-stage interventions in preclinical AD models merits further investigation into the protective potential of FUS in asymptomatic AD. The demonstrated benefit of using FUS in models of late-stage AD motivates the present study’s attempt to prevent AD via the same neuroprotective and remedial mechanisms, albeit at an earlier intervention timepoint prior to symptom onset. An effective preventative therapeutic should not only reduce the accumulation of amyloid and tau, but must also demonstrate cognitive improvement. Successful realization of both of these aims is necessary to improve the current standard of AD therapeutics and demonstrate translational relevance and progress. Thus, the objective of this study is to characterize the protective role of FUS-BBBO in asymptomatic AD for delaying the onset of both cognitive and pathological symptoms in AD.

## 2. Materials and methods

### 2.1. Study design

This study was designed to harness the previously demonstrated efficacy of FUS-BBBO in effecting benefits in late-stage AD for early-stage intervention and prevention *in vivo* (Burgess et al., 2014; Leinenga and Götz, 2015; Karakatsani et al., 2019; Blackmore et al., 2023). The safety of the treatment paradigm and FUS parameters applied here has been confirmed by previous studies (Samiotaki et al., 2012; Olumolade et al., 2016). This study aims to expand on the previously characterized effects of FUS in AD by evaluating the efficacy of FUS for protecting against anxiety, memory, and learning deficits as well as AD-associated protein accumulation in early-stage AD.

### 2.2. Animal use

**FIGURE 1:**
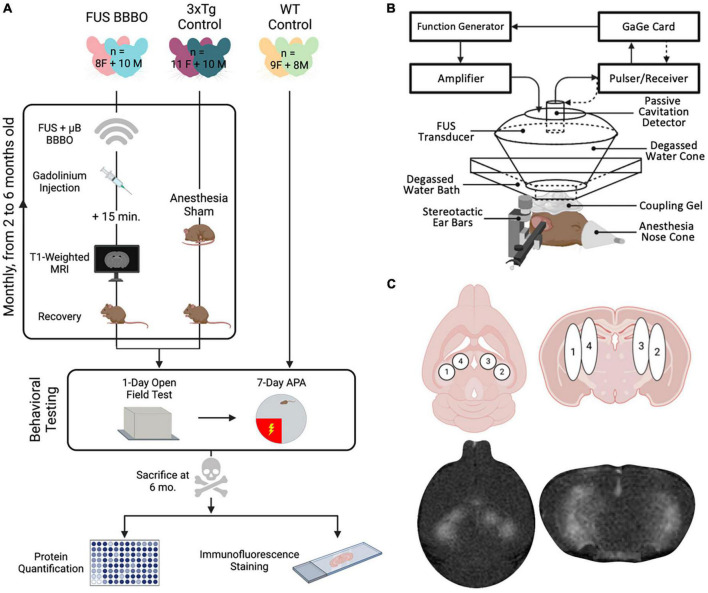
Experimental overview and FUS parameters. **(A)** Experimental procedure timeline including all six cohorts of mice. Experiments included monthly sonications or sham condition, followed by behavioral testing and finally protein quantification and histology. **(B)** Focused ultrasound setup using a spherical transducer. **(C)** FUS targeting, consisting of four sonication spots bilaterally covering the hippocampus. Figures were generated in BioRender.com.

All animals were housed and handled in compliance with Columbia University’s Institutional Animal Care and Use Committee under protocol #AC-AABG4559. A total of 20 female and 20 male triple transgenic (3xTg) mice were used for the present study (The Jackson Laboratory, MMRRC stock #34830) (Oddo et al., 2003). 3xTg mice were generated by Oddo et al. (2003) by coinjecting transgenes encoding human APP_Swe_ and tau_P301L_ into a PS1_M146B_ knockin mouse. These mutations give rise to a mouse model that exhibits both amyloid and tau pathology, in addition to synaptic dysfunction (Oddo et al., 2003). Ten female, and ten male 3xTg mice received 5 monthly FUS-BBBO sessions beginning at 2 months of age, and the remaining 10 of each sex were reserved as untreated controls. The animals were treated and tested in three cohorts to amass the final set of 40 animals. Each cohort was comprised of an equal distribution of male or female, and experimental or control group animals in each cohort to control for any between-cohort biases. Control cohorts were anesthetized for a brief period monthly, on schedule with experimental group FUS-BBBO sessions, to control for any confounding effects of anesthesia. After the 5 months of FUS-BBBO or anesthesia-only sessions, all animals underwent behavioral testing, and were then euthanized by cardiac perfusion for protein quantification and immunohistological staining (Figure 1A). Animal weights were taken each month to monitor health throughout the long-term study (Supplementary Figure 2A).

### 2.3. Polydisperse microbubbles

In-house made lipid-shelled, gas-filled, polydisperse microbubbles were made as previously described (Wang et al., 2014). Briefly, 1, 2-disteraroyl-sn-glycero-3-phosphocholine (DSPC) (Avanti Polar Lipids Inc., Alabaster, AL, USA) and polyethylene glycol 2000 (Avanti Polar Lipids Inc., Alabaster, AL, USA) were combined at a 9:1 molar ratio. These lipids were then dissolved at 2 mg/ml in a filtered solution of 80% PBS, 10% glycerol, and 10% propylene glycol. The solution was submerged in a >60°C water bath and subjected to degassing and sonication for ≥1 h until all particles were dissolved. A total of 2 ml aliquots were then made and stored at 4°C until activation. The microbubbles were activated prior to use by vacuuming excess air from a sealed vial, filling the headspace with perfluorobutane gas, and agitating the vial with a VialMix (Lantheus Medical Imaging, N. Billerica, MA, USA) shaker to form polydisperse microbubbles.

### 2.4. Focused ultrasound blood–brain barrier opening

Focused ultrasound-mediated BBBO was performed in FUS-BBBO cohorts using previously established safe parameters. A single-element, concave FUS transducer (center frequency: 1.5 MHz, focal length: 60 mm, diameter: 60 mm; Imasonic, France) was operated at a Peak Negative Pressure of 450 kPa with a pulse repetition frequency of 4 Hz (Samiotaki et al., 2012; Olumolade et al., 2016). An additional single-element transducer (V320, frequency: 7.5 MHz, focal length: 52 mm, diameter: 13 mm; Olympus NDT, Waltham, MA, USA) was confocally aligned with the FUS transducer focus and used for passive cavitation detection to monitor microbubble activity in real-time during sonication. The transducers were fixed to a 3D positioning system for targeting. For each BBBO session the subject was anesthetized with 3–4% isoflurane until induction was confirmed by lack of response to toe pinch. Subjects’ heads were stabilized using a stereotactic apparatus and anesthesia was maintained via a nose cone delivering 1.5–2% isoflurane for the duration of the session. Subjects’ heads were shaved with electric clippers and any remaining hair was removed with depilatory cream. Degassed ultrasound coupling gel was placed on the subject’s head, on which a bath of degassed water was set. The transducer was submerged in the water bath and targeting was performed to center the transducer over the lambdoid structure. The FUS setup is shown in Figure 1B. The transducer was then moved, using the 3D positioning system, to each of the four targets bilaterally covering the hippocampus (Figure 1C), to record 10 s of control pulses at each spot prior to the injection of microbubbles. A total of 5 μl activated in-house made, lipid-shelled, polydisperse microbubbles were then combined with 45 μl saline to make a solution of 8 × 10^8^ μB/ml. The bolus injection of microbubbles was then introduced via the tail vein, and the transducer was activated to sonicate for 60 s at each of the four target spots. The microbubble cavitation activity was recorded for cavitation dose quantification.

### 2.5. Magnetic resonance imaging

Each mouse received an intraperitoneal injection of 0.2 ml gadomide (Gd) contrast agent (Omniscan, GE Healthcare, Chicago, IL, USA). The contrast agent was allowed to diffuse and the mouse was anesthetized with 3–4% isoflurane until unresponsive to toe pinch. The mouse was set inside the MRI’s vertical bore (Bruker Ascend™ 400 MHZ WB 9.4T), and exactly 15 min after the Gd was injected a T1-weighted 2D FLASH sequence (TR: 230 ms, TE: 3.3 ms, Flip angle: 70°, Averages: 6, FOV: 25.6 mm × 25.6 mm, Matrix size: 256 × 256, Slice thickness: 0.4 mm, Resolution: 0.1 mm × 0.1 mm, Scan time: 5 min) was initiated to acquire MR images of the brain in both axial and coronal orientations. These contrast-enhanced images were used to confirm BBB targeting and for later BBBO quantification.

### 2.6. Behavioral testing

All mice were handled for 30–60 s daily for 1 week prior to undergoing behavioral testing. Twenty-four hours before the first day of testing the mice were handled in the behavioral room to acclimate them to the new space. On the first day of testing, mice were housed in their cages for 30 min in the behavior room for habituation. The habituation paradigm was conducted to reduce testing room anxiety as recommended by Willis et al. (2017). Subject order was randomized between treatment days to interleave treatment and control, as well as male and female cohorts, to mitigate any confounding effects of treatment order.

#### 2.6.1. Open field testing

The open field behavioral test was performed as previously described. A 40 cm × 40 cm × 29.5 cm (L × W × H) opaque arena with an open top was centered under a Basler acA1300-60gm camera (Basler, Ahrensburg, Germany) inside of a noise canceling room (MDL 4848 S, Whisper Room Inc., Knoxville, TN, USA). An 8 cm-wide peripheral zone was defined along the edge of the arena (64% of the arena), and the remaining inner region (576 cm^2^) was defined as a central zone (36% of the arena area) (Supplementary Figure 4A). Each subject was placed mid-way along one of the arena walls, facing the wall at the start of the trial. The mouse was then allowed to explore the arena for 20 min, while Ethovision XT tracking software (Noldus Information Technology, Leesburg, VA, USA) recorded the total distance traveled, and the time elapsed in inner and peripheral zones in 5-min intervals. The arena was cleaned with 70% ethanol between subject trials.

#### 2.6.2. Active place avoidance

The active place avoidance (APA) behavioral test was performed as previously described (Willis et al., 2017). A 28 in. × 28 in. rotating shock grid was centered beneath a Basler Ace. Monochrome IR-sensitive GigE camera with a transparent cylindrical enclosure measuring 60 cm in diameter (Maze Engineers, Skokie, IL, USA). The table was enclosed within a noise canceling room (MDL 4848 S, Whisper Room Inc., Knoxville, TN, USA) to control light, sound, and air circulation during testing. Four unique black and white patterns measuring 8.5” × 11” were posted at the four corners of the room, dividing the circular testing arena into four quadrants (Supplementary Figure 4B). The shock quadrant was defined as north, the opposite as south, and the adjacent two quadrants as west and east, respectively. The APA trial was comprised of three phases: a 1-day habituation trial, 5 days of spatial learning and a 1-day reversal trial.

First, a habituation trial was performed 24 h before the first day of shock to acclimate the animals to the rotating metal grid. Each animal was placed in the south quadrant of cylindrical enclosure atop the table rotating counterclockwise at 1 rpm and was allowed to explore the arena freely in the absence of shock. The distance traveled and amount of time elapsed in each quadrant was recorded using Ethovision XT tracking software (Noldus Information Technology, Leesburg, VA, USA) (Supplementary Figure 5). After the 20-min habituation trial the mouse was removed from the arena and allowed to recover in an isolated enclosure. The arena was cleaned with 70% ethanol between trials.

Twenty-four hours after the habituation trial, on the first of the 5-day spatial learning paradigm each subject was placed in the south quadrant of the circular arena rotating counterclockwise at 1 rpm. Each time the subject entered the shock zone, the grid delivered a 500 ms, 60 Hz 0.5 mA foot shock. Further shocks were delivered at 1.5-s intervals until the animal exited the shock zone. Each re-entry into the shock zone triggered an additional series of shocks persisting until the subject exited the shock zone. The total distance traveled by the subject, the trial time elapsed in each quadrant, the number of shocks delivered, and the maximum inter-shock interval (maximum time of avoidance) were recorded in 5-min intervals for the duration of the 20-min trial using Ethovision XT tracking software (Noldus Information Technology, Leesburg VA, USA) (Bahník and Stuchlík, 2015; Willis et al., 2017). At the end of the trial the subject was allowed to recover in an isolated enclosure and the arena was cleaned with 70% ethanol between trials. This paradigm was repeated for 5 total days.

After the fifth day of spatial learning, the shock and opposite quadrants were switched for the reversal trial. The south quadrant was made the new shock zone, and each subject was placed into the north quadrant to begin the trial. Identical spatial cues and shock conditions were used, and again the total distance traveled by the subject, the fraction of trial time elapsed in each quadrant, the number of shocks delivered, and the maximum inter-shock interval were recorded in 5-min intervals for the duration of the 20-min trial. Each animal was removed from the testing arena and allowed to recover in isolation at the end of the trial.

### 2.7. Protein quantification

Each subject was euthanized by heavy induction with isoflurane (4–5%) and cardiac perfusion with sterile saline chilled to 4°C. The hippocampus was dissected from the rest of the brain and flash frozen on dry ice. The frozen tissue was weighed and combined with T-PER solution [1:1,000 Halt Protease and Phosphatase Inhibitor Cocktail (ThermoFisher Scientific, Cat#78446), in T-PER Protein Extraction Reagent (ThermoFisher Scientific, Cat#78510)] at a concentration of 0.1 mg/μl. The tissue was homogenized in the solution on wet ice, then centrifuged for 30 min at 15,000 *g* at 4°C. The supernatant, containing soluble protein, was collected, strained through a 70 μm filter, and stored at −80°C until quantification.

The total BCA protein content was measured for each mouse using the Pierce™ BCA Protein Assay Kit (ThermoFisher Scientific, Cat#23225) and used for normalization of the measured quantity of AD protein targets.

The amount of human Aβ40, Aβ42, and total tau was measured using the multiplexed bead-based ProcartaPlex Simplex kits for each target. The samples were processed in duplicates, the mean of the duplicate reads was taken, and this mean was normalized by the BCA measurement for each sample to give a normalized concentration of each of the three targets for each subject.

### 2.8. Histological analysis

#### 2.8.1. Immunofluorescence preparation

Each subject was euthanized by heavy induction with isoflurane (4–5%) and cardiac perfusion with sterile saline chilled to 4°C. The brain was fixed in 4% PFA at 4°C for 48 h, then one brain from each 3xTg cohort was moved to a 30% sucrose solution for 24–48. Once removed from the sucrose solution the brain was embedded in O.C.T. (Tissue-Tek, #4583). The brains were cryosectioned coronally into 35-μm sections on a Leica CM1850 cryostat (Leica Biosystems, Deer Park, IL, USA). The sections were stored in 0.01% sodium azide until immunohistological staining.

Coronal sections including the targeted hippocampus were selected from each 3xTg cohort for immunofluorescence staining of hyper-phosphorylated tau. The sections were washed in 1× PBS three times for 5 min each. Next, the sections were blocked with 5% NDS in 0.3% Triton X-100 for 20 min, then moved to a solution of 1:100 mouse seroblock (FisherScientific, #NC0286307) and 5% NDS in 0.3% Triton X-100 for 10 min. Next the sections were incubated with the primary HT7 (FisherScientific, #ENMN1000) antibody at a concentration of 1:100 in 5% NDS and 0.3% Triton X-100. The sections were incubated overnight at 4°C in the primary antibody solution. The next day the sections were washed in 0.3% Triton X-100 three times for 10 min each. The sections were then incubated in a secondary antibody solution containing donkey α mouse AF488 at a concentration of 1:1,000 (Abcam, ab150105) for 2 h at room temperature. Finally, the sections were washed three times in 1× PBS for 10 min each, mounted on glass slides, coverslipped with DAPI mounting medium (Abcam, ab104139) and imaged at 10× with a fluorescent microscope.

#### 2.8.2. Hematoxylin and eosin preparation

One mouse from each cohort was perfused as described above, the brain was dissected and transferred to 4% paraformaldehyde for 24–48 h, and then embedded into paraffin. The brain was then sectioned coronally into 5-μm sections. The sections were then stained with hematoxylin and eosin (H&E) and imaged on a brightfield microscope.

### 2.9. Statistical analysis

**FIGURE 2:**
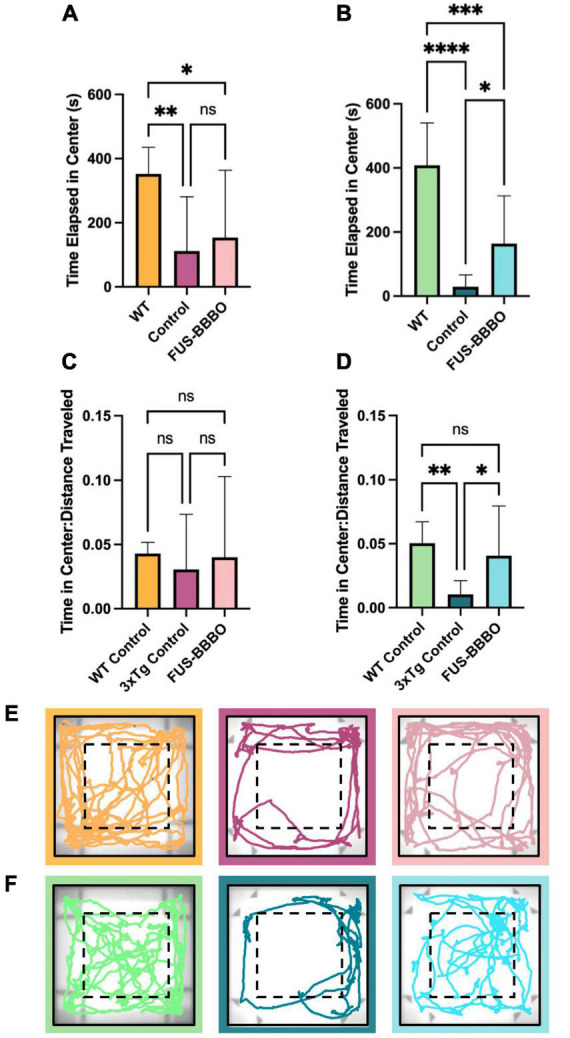
Open field test for anxiety testing. The time elapsed in the center portion of the arena is shown for females **(A)** and males **(B)**. The time elapsed in the center, normalized by the distance traveled by each mouse is shown for females **(C)** and males **(D)** to give a locomotion-independent metric of time spent in the center of the arena. Representative traces of the final 5 min of the OFT are shown for female **(E)** and male **(F)** cohorts. One-way ANOVA tests with multiple comparisons were performed to determine statistically significant differences between groups. *P ≤ 0.05; **P ≤ 0.01; ***P ≤ 0.001; ****P ≤ 0.0001.

**FIGURE 3:**
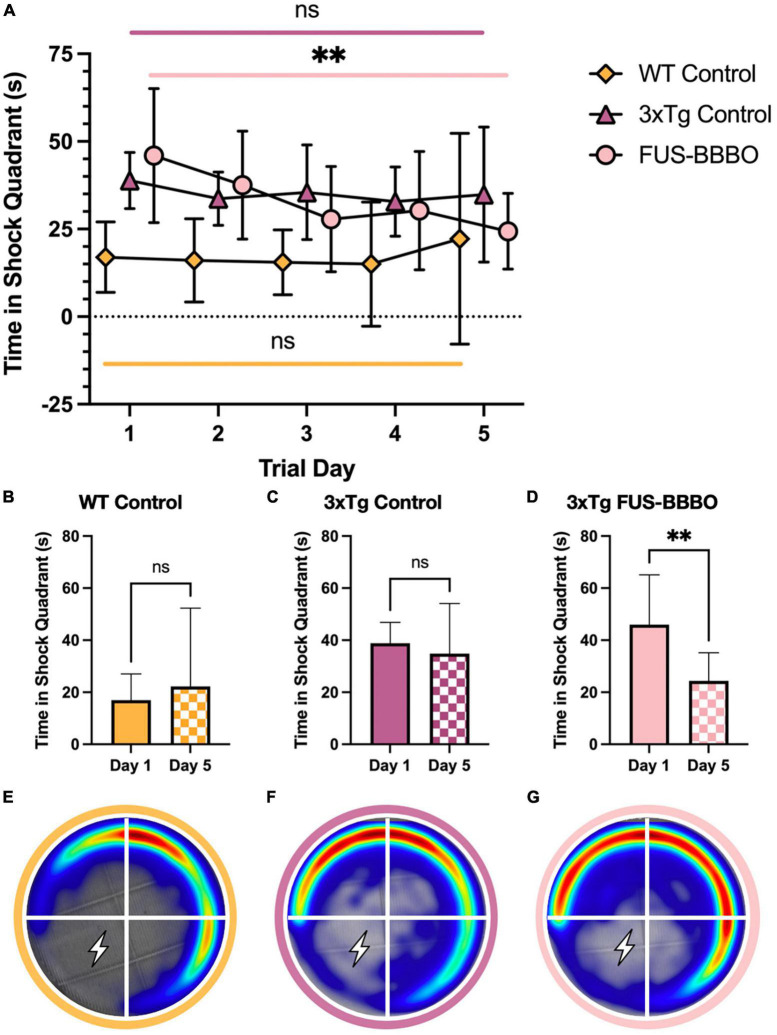
Focused ultrasound-induced blood–brain barrier opening improves female performance in active place avoidance test. **(A)** The time elapsed in the shock quadrant over the 5 training days is shown for each female cohort. The statistical difference between the first and fifth training day is evaluated with a paired *t*-test. The time elapsed in the shock quadrant on day 1 and 5 of the APA training is shown for the WT **(B)**, 3xTg control **(C)**, and FUS-BBBO **(D)** females. Averaged heat maps for female WT **(E)**, 3xTg control **(F)**, and FUS-BBBO **(G)** are shown. **P ≤ 0.01.

**FIGURE 4:**
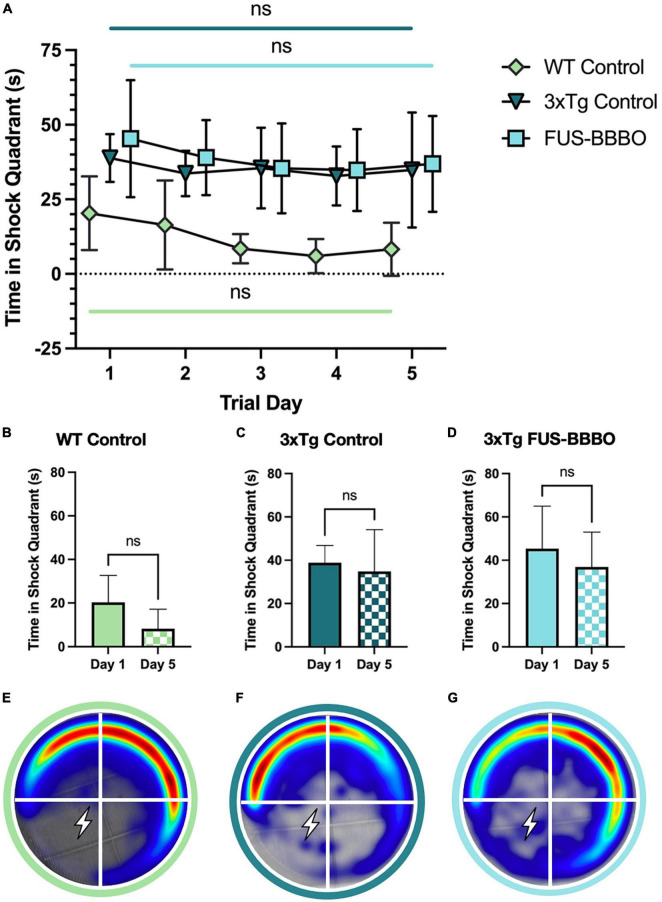
Focused ultrasound-induced blood–brain barrier opening does not significantly affect male performance in active place avoidance test. **(A)** The time elapsed in the shock quadrant over the 5 training days is shown for each male cohort. The statistical difference between the first and fifth training day is evaluated with a paired *t*-test. The time elapsed in the shock quadrant on day 1 and 5 of the APA training is shown for WT **(B)**, 3xTg control **(C)**, and FUS-BBBO **(D)** males. Averaged heat maps for male WT **(E)**, 3xTg control **(F)**, and FUS-BBBO **(G)** cohorts are shown.

**FIGURE 5:**
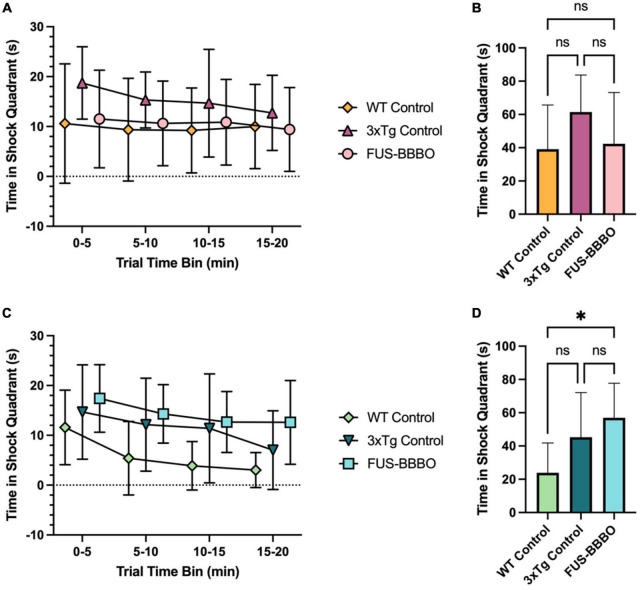
Focused ultrasound-induced blood–brain barrier opening improves female performance in active place avoidance reversal trial. **(A)** The time elapsed in the shock quadrant over the course of the 20-min APA reversal trial is shown in 5-min time bins for each female cohort. **(B)** The time elapsed in the shock quadrant for each female cohort over the entire reversal trial is shown. **(C)** The time elapsed in the shock quadrant over the course of the 20-min APA reversal trial is shown in 5-min time bins for each male cohort. **(D)** The time elapsed in the shock quadrant for each male cohort over the entire reversal trial is shown. Statistically significant differences are determined by one-way ANOVA tests with multiple comparisons for panels **(B,D)**. *P ≤ 0.05.

**FIGURE 6:**
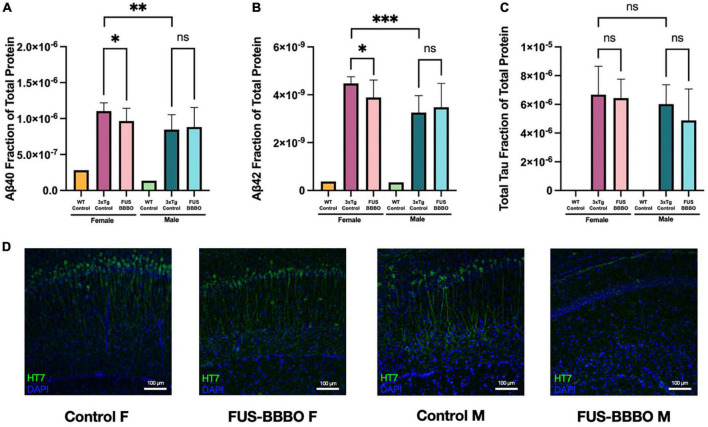
Focused ultrasound-induced blood–brain barrier opening prevents the accumulation of AD-associated proteins in females. The mean total-protein normalized concentration of amyloid-β40 **(A)**, amyloid-β42 **(B)**, and total tau **(C)** is shown for each 3xTg cohort. Quantification of each target protein in WT tissue was included as a negative control and was not included in statistical tests. **(D)** Representative immuofluorescence images of tau (HT7) are shown for each 3xTg cohort. Statistical significance is determined by pairwise unpaired *t*-tests. *P ≤ 0.05; **P ≤ 0.01; ***P ≤ 0.001.

Male and female mice were analyzed separately due to known differences between sexes in AD pathology progression and cognitive deficits (Carroll et al., 2010; Pairojana et al., 2021). Male and female subjects were interleaved for FUS-BBBO sessions to decrease potential bias and batch effects. Similarly, the order of behavioral testing was randomized and changed daily to prevent any potential bias resulting from subject testing order. The exclusion criteria for behavioral analysis is provided in Supplementary Figure 3. One-way ANOVA tests with multiple comparisons were used to determine statistical differences between all groups by comparing each group’s mean with the mean of every other for Figures 2A–D, 5B, D. Statistical differences within groups at two different timepoints (day 1 and 5 of APA training) were determined using paired *t*-tests for Figures 3B–D, 4B–D. Pairwise unpaired *t*-tests were used to evaluate significant differences between different groups, namely female 3xTg control vs. FUS-BBBO groups, male 3xTg control vs. FUS-BBBO groups, and female vs. male 3xTg control groups in Figures 6A–C. Statistical analysis was performed using GraphPad Prism (Version 9.2.0 for macOS, GraphPad Software, San Diego, CA, USA). All error bars indicate standard deviation and statistical significance is defined as follows: *P ≤ 0.05; **P ≤ 0.01; ***P ≤ 0.001; ****P ≤ 0.0001.

## 3. Results

### 3.1. FUS-BBBO decreases anxiety in 3xTg females as measured by time elapsed in center region of open field test

No significant difference in locomotion was detected between control and FUS-BBBO 3xTg female cohorts as measured by the average distance traveled over a 20-min open field test (OFT) trial (*P* = 0.9898, one-way ANOVA with multiple comparisons) (Supplementary Figure 1A). This result confirms previous findings which demonstrate no difference in locomotion following long-term repeated exposure to FUS-BBBO (Olumolade et al., 2016). WT females demonstrated significantly greater locomotion compared to both control and FUS-BBBO 3xTgs (*P* < 0.0001 and *P* < 0.0001, respectively, one-way ANOVA with multiple comparisons) (Supplementary Figure 1A).

The amount of time elapsed in the center zone of the OFT arena is a measure of an animal’s propensity for exploration, or level of anxiety. WT females spent significantly more time in the center of the arena than control 3xTg females (Figure 2A) (*P* = 0.0075, one-way ANOVA with multiple comparisons). Interestingly, there was a less significant difference between the amount of time WT and FUS-BBBO 3xTg females spent in the center of the arena (*P* = 0.0452, one-way ANOVA with multiple comparisons) and although there was no significant difference between control and FUS-BBBO 3xTg females (*P* = 0.8434, one-way ANOVA with multiple comparisons), the average amount of time that FUS-BBBO females spent in the center was 37.42% greater than the 3xTg controls on average. Dividing the time elapsed in the center of the arena by the distance traveled by each animal gives a normalized metric for quantifying the relative exploratory behavior of each cohort. Both WT and FUS-BBBO 3xTg female cohorts demonstrated trending yet not significantly increased normalized time elapsed in the center of the arena relative to 3xTg controls (Figure 2C) (*P* = 0.8019 and *P* = 0.8828, respectively, one-way ANOVA with multiple comparisons). FUS-BBBO 3xTg and WT females normalized time in center was not significantly different (*P* = 0.9904, one-way ANOVA with multiple comparisons).

By all of these metrics, FUS-BBBO 3xTg females demonstrate reduced levels of anxiety, more closely resembling WT performance, compared to untreated 3xTg females.

### 3.2. FUS-BBBO decreases anxiety in 3xTg males as measured by time elapsed in center region of open field test

Interestingly, FUS-BBBO 3xTg males demonstrated significantly higher locomotion than untreated 3xTg control animals (*P* = 0.0028, one-way ANOVA with multiple comparisons) (Supplementary Figure 1B). This increased locomotion more closely resembles the activity level of WT males, which traveled significantly greater distance on average compared to both control and FUS-BBBO 3xTgs (*P* < 0.0001 and *P* < 0.0001, respectively, one-way ANOVA with multiple comparisons) (Supplementary Figure 1B).

In males, WT mice spent more time in the center of the arena than the 3xTg controls and FUS-BBBO cohorts (*P* < 0.0001 and *P* = 0.0004, respectively, one-way ANOVA with multiple comparisons), and the FUS-BBBO cohort spent significantly more time in the center than 3xTg controls (*P* = 0.0388, one-way ANOVA with multiple comparisons) (Figure 2B). The increased time elapsed in the center by FUS-BBBO males represents a 463.02% increase compared to untreated 3xTg control males. WT and FUS-BBBO 3xTg cohorts both had significantly greater normalized time in the center compared to 3xTg controls (*P* = 0.0085 and *P* = 0.0384, respectively, one-way ANOVA with multiple comparisons), and the difference between WT and FUS-BBBO 3xTg was not significant (*P* = 0.7043, one-way ANOVA with multiple comparisons) (Figure 2D).

The increased time elapsed in the center of the OFT arena by FUS-BBBO compared to control 3xTg cohorts, mimicking WT-like behavior, indicates that FUS-BBBO effectively prevents the progression of AD-associated anxiety in both male and female 3xTg cohorts. Representative OFT traces are shown for females and males in Figures 2E, F, respectively.

### 3.3. FUS-BBBO improves spatial learning in female 3xTgs in active place avoidance paradigm

The amount of time elapsed by each cohort in each of the four testing quadrants during the APA habituation trial demonstrates no bias in the testing arena and is shown in Supplementary Figure 5.

In the 5-day APA shock trial paradigm, the female FUS-BBBO 3xTg cohort demonstrated improved spatial memory for the shock zone compared to age-matched, untreated control cohorts. FUS-BBBO 3xTg females spent less time in the shock quadrant than 3xTg controls on days 3, 4, and 5 of the trial (Figure 3A). Although there is no significant difference between any of the groups on day 5 (*P* > 0.05, one-way ANOVA with multiple comparisons), FUS-BBBO 3xTg females spent 30.03% less time in the shock quadrant compared to untreated 3xTg female controls. Additionally, the FUS-BBBO 3xTg females show a significant, 46.96% reduction in the time elapsed in the shock quadrant from day 1 to day 5 (*P* = 0.0067, paired *t*-test) (Figure 3D), demonstrating significant learning. The difference between day 1 and day 5 performance is not significant for either the WT or control 3xTg female cohorts (Figures 3B, C) (*P* = 0.5209 and 0.5340, respectively, paired *t*-test). Averaged heat maps for each female cohort over the APA arena are shown in Figures 3E–G.

These findings indicate that in 6-month-old females, FUS-BBBO slowed or prevented the progression of spatial memory deficits that inhibited learning and shock avoidance in the control 3xTg cohort.

### 3.4. FUS-BBBO did not significantly affect the performance of 3xTg males in active place avoidance paradigm

There was no significant difference in the average time elapsed in the shock quadrant by FUS-BBBO compared to untreated control 3xTg male cohorts over the 5-day training paradigm (Figure 4A). FUS-BBBO 3xTg males demonstrated an 18.65% reduction in the time elapsed in the shock quadrant between days 1 and 5, while the control 3xTg cohort showed only a 10.33% reduction (Figures 4C, D). However, none of the 6-month-old male cohorts, WT, control 3xTg or FUS-BBBO 3xTg, demonstrated statistically significant improvement over the 5 training days (*P* = 0.1076, *P* = 0.5340, and *P* = 0.3186, respectively, paired *t*-test) (Figures 4B–D). Despite the lack of significant learning, the WT male cohort outperformed both 3xTg cohorts, spending less time in the shock zone on all 5 training days. No significant differences were detected between 3xTg control and FUS-BBBO cohorts on any of the training days. Average heat maps for the cohorts are shown in Figures 4E–G.

Focused ultrasound-induced blood–brain barrier opening may have preserved some cognition and learning in 6-month-old males given the greater learning difference in FUS-BBBO compared to control 3xTg males between day 1 and 5, despite the lack of observed statistical significance.

### 3.5. FUS-BBBO improves neural plasticity via active place avoidance reversal task in 6-month-old female 3xTg mice

In the 1-day APA reversal trial female FUS-BBBO 3xTg mice demonstrated enhanced performance compared to the age-matched, untreated, female 3xTg controls. The time elapsed in the shock quadrant is shown in 5-min time bins over the course of the 20-min reversal trial in Figure 5A. The differences between the three female cohorts are not significant in any time bin by one-way ANOVA with multiple comparisons, however, the FUS-BBBO females performed comparably to the WT cohort, and the untreated 3xTg controls had the worst performance, spending the most time in the shock quadrant in every time bin. This trend persists when quantifying the total time elapsed in the shock quadrant over the 20-min trial (Figure 5B). By this metric also, the performance of the FUS-BBBO 3xTg females most closely resembled that of the WT females with no significant difference detected between the two (*P* = 0.9652, one-way ANOVA with multiple comparisons). Although the differences between the three cohorts were not significant by one-way ANOVA with multiple comparisons, the FUS-BBBO 3xTg cohort spent 31.01% less time in the shock quadrant over the entire trial than the control 3xTg cohort.

The similarity between the performance of the WT and FUS-BBBO 3xTg females indicates that FUS-BBBO effectively preserved the neural plasticity that is lost with disease progression in the untreated female 3xTg control mice.

### 3.6. FUS-BBBO does not affect neural plasticity via active place avoidance reversal task in 6-month-old male 3xTg mice

Focused ultrasound-induced blood–brain barrier opening did not significantly affect the performance of 3xTg male mice in the APA reversal trial compared to untreated, 3xTg controls (Figures 5C, D). The WT male mice spent less time in the shock quadrant over the entire reversal trial than both the FUS-BBBO and control 3xTg cohorts (*P* = 0.0125 and *P* = 0.1314, respectively, one-way ANOVA with multiple comparisons), however, there was no significant difference in the total amount of time elapsed in the shock quadrant between the FUS-BBBO and control 3xTg male cohorts (*P* = 0.4829, one-way ANOVA with multiple comparisons), indicating that FUS-BBBO did not significantly affect the performance of 6-month-old male 3xTg mice.

### 3.7. FUS-BBBO prevents the accumulation of amyloid-β and tau in female 3xTg mice

Focused ultrasound-induced blood–brain barrier opening 3xTg females presented significant 12.54 and 13.05% reductions in Aβ40 (Figure 6A) (*P* = 0.0499, unpaired *t*-test) and Aβ42 (Figure 6B) (*P* = 0.0279, unpaired *t*-test) compared to untreated 3xTg control females. Although not statistically significant, FUS-BBBO gave rise to a 3.57% decrease in total tau compared to untreated control females (Figure 6C) (*P* = 0.7466, unpaired *t*-test).

### 3.8. FUS-BBBO prevents the accumulation of tau in male 3xTg mice

There was no significant difference between FUS-BBBO and control 3xTg males for Aβ40 or Aβ42 (*P* = 0.7724 and *P* = 0.6268, respectively, unpaired *t*-test) (Figures 6A, B). However, FUS-BBBO gave rise to a trending 18.98% reduction in total tau accumulation compared to control 3xTg males (*P* = 0.2410, unpaired *t*-test) (Figure 6C).

WT female and male mice (*n* = 1 / gender) were included in the protein quantification assay as negative controls for each of the three protein targets. Representative immunohistochemistry images are shown for each 3xTg cohort in Figure 6D.

### 3.9. Monthly FUS-BBBO does not negatively affect the health of 3xTg mice

The weights of all 3xTg subjects were taken every month prior to each of the five FUS-BBBO sessions. These weights were plotted as a function of time and are shown in Supplementary Figure 2A. The growth rates of the male and female FUS-BBBO cohorts did not deviate significantly from their untreated, age-matched, control littermates. The only month that demonstrated a significant difference by unpaired *t*-test was month 2 of treatment, at 3 months of age, when the average control female cohort weight was greater than that of the FUS-BBBO females (*P* = 0.0478). Throughout the remainder of the trial months the FUS-BBBO male and female average weights were not significantly different than the respective control cohort.

Additionally, one mouse from each 3xTg cohort was perfused and processed for H&E staining. No red blood cell extravasation or damage was detected in the H&E images from each cohort, suggesting that no damage was induced by repeated FUS-BBBO over the 5-month treatment period (Supplementary Figures 2B–E).

Taken together, these data demonstrate that early, repeated intervention with non-invasive FUS-BBBO is capable of preventing AD-associated anxiety, spatial memory and reversal learning deficits, as well as protein accumulation *in vivo* without compromising health. A summary of these results is provided in Table 1. These findings offer a promising option for translation to human populations at high risk of developing AD.

**TABLE 1:** Study results summary.

		Females	Males
Anxiety (OFT)		Improved	Improved
Spatial memory (APA)		Improved	No change
Reversal memory (APA)		Improved	No change
AD pathology	Aβ40	Decreased	No change
	Aβ42	Decreased	No change
	Total tau	Decreased	Decreased

Summary of test results for female and male FUS-BBBO 3xTg cohorts compared to age- and gender-matched, control 3xTg cohorts.

## 4. Discussion

This study demonstrates the potential utility of early, repeated FUS-BBBO as a non-invasive preventative therapeutic intervention for the psychiatric, cognitive and pathological symptoms of AD.

Subject activity in the open field test is inversely related to their level of anxiety (Crawley, 1985). Although the average locomotion, as measured by distance traveled over the course of the OFT, did not differ significantly between control and FUS-BBBO 3xTg females, there was a clear increase in FUS-BBBO 3xTg male locomotion that more closely resembles WT activity levels. This increase indicates a positive change in locomotive behavior in male 3xTg mice after repeated FUS-BBBO.

It has been shown that with age, 3xTg mice exhibit a heightened vulnerability to novelty, or intensified anxiety, as measured by various metrics in the open field behavioral paradigm (Roda et al., 2020). One of these variables, the amount of time spent in the brightly lit, open, center portion of the area, is indicative of an animal’s general level of anxiety and propensity for exploration (Guest, 2019). Conversely, a preference for the periphery of the arena near the walls is a display of anxiety-like behavior (Guest, 2019). Given that anxiety is not only highly prevalent in AD populations at 39%, but has also been identified as a risk factor for later AD diagnoses, the ability to prevent the progression and expression of anxiety-like behaviors is of great interest (Zhao et al., 2016; Santabárbara et al., 2019; Mendez, 2021). Effective anxiety reduction in this population will relieve some of the economic and clinical burdens imposed by AD. Thus, the finding that both female and male FUS-BBBO 3xTg cohorts exhibit increased time in the center of the arena by both measured and normalized metrics (Figures 2A–D) is a promising demonstration of the potential for FUS-BBBO to alleviate this neuropsychiatric aspect of AD. There have been preliminary studies demonstrating anxiety amelioration in progressed models of AD, but this study goes further to present the sex-specific, protective potential of FUS-BBBO for AD-associated anxiety in an early intervention paradigm (Shen et al., 2020).

The 5-day APA paradigm offers a metric for quantifying spatial, short-term and long-term memory capabilities (Willis et al., 2017). Over the five training days performed in the present study, the female FUS-BBBO 3xTg cohort outperformed the 3xTg control group on the final 3 days, spending less time in the shock quadrant, and nearing the WT-level of performance by the final day. Compromised long-term memory is a well-established hallmark of AD (Stopford et al., 2012). The time elapsed in the shock quadrant over multiple training days demonstrates the relative long-term learning and memory capabilities of each cohort. The significant 30.03% difference detected between the FUS-BBBO female cohort’s first and final day of APA training demonstrates substantial learning over the course of the trial. This learning is not evident in the control 3xTg cohort, which does not demonstrate a significant difference in the amount of time elapsed in the shock quadrant between the first and fifth shock trial day. Although the WT cohort also does not demonstrate significant learning, this cohort consistently spends a minimal amount of time in the shock quadrant on all 5 training days, setting a gold standard for uninhibited performance in the task. The trend toward WT performance in FUS-BBBO females indicates that their treatment has prevented the cognitive decline in spatial memory exhibited by the untreated control 3xTg cohort.

The lack of statistically significant differences between male FUS-BBBO and control 3xTg cohorts indicates that FUS-BBBO had no significant effect on the spatial memory of male 3xTg mice at 6 months of age. Both 3xTg male cohorts performed worse than the male WT cohort, spending more time in the shock quadrant throughout the 5-day training period, although the FUS-BBBO males demonstrated greater learning, with a 18.65% improvement from the first to final training day compared to only a 10.33% improvement in untreated 3xTg males. It is likely that the lack of significant differences between FUS-BBBO and control 3xTg males is due to the lack of disease progression at this early age in male mice relative to females in this AD model. While female 3xTg mice have already accumulated significant pathology and behavioral deficits by 6 months of age, the progression of the aforementioned is delayed in 3xTg males, allowing less room for improvement at this early timepoint with a preventative intervention (Clinton et al., 2007).

While spatial and long-term memory improvements have been demonstrated following FUS in aged and progressed AD models of a single sex, this study offers insight into the effects of early intervention, and also compares the male vs. female response to this intervention.

Working memory, which refers to the information that can be temporarily stored and directly applied to perform cognitive tasks, has been shown to decline with AD progression (Stopford et al., 2012; Cowan, 2014). The temporal plot showing the time each cohort elapsed in the shock zone over the 20-min reversal trial in Figure 5 illustrates the strength of a given group’s working memory for the location of a novel shock zone. The reduction in the number of shocks delivered to FUS-BBBO females over the course of the trial indicates improved working memory and propensity for reversal learning compared to control mice (Figures 5A, B). The FUS-BBBO 3xTg female’s WT-mimicking performance in the reversal trial indicates that FUS-BBBO has preserved both neural plasticity and working memory in these subjects. FUS-BBBO cohorts demonstrate enhanced neural plasticity by first, quickly un-learning their previously memorized spatial map of the shock zone relative to spatial cues, and second, more robustly re-learning a new spatial map that includes a novel shock zone location. This improvement in treated female mice demonstrates that FUS-BBBO application has effectively prevented the deficits accumulated by the untreated control 3xTg mice. In males, the lack of significant differences between the FUS-BBBO and untreated control 3xTg mice is likely attributable to the lack of pathological accumulation at 6 months of age. In the absence of significant accrued deficits, there will be little opportunity for improvement by a preventative therapeutic. Thus, as there is no significant difference between male WT and the 3xTg control performance in the reversal trial, and no significant difference between the FUS-BBBO and control male 3xTg cohorts, we can infer that there was no significant deficit in 6-month-old 3xTg males that could have been prevented by FUS-BBBO.

Although the role of AD-associated pathologies in AD onset as a causal agent is uncertain, it remains evident that the accumulations of Aβ and tau contribute to the progression of neurodegeneration and synaptic dysfunction, which give rise to cognitive deficits and clinical symptoms in AD (Ballatore et al., 2007; Crews and Masliah, 2010). Additionally, females are disproportionately affected by AD, with three female for every two male AD diagnoses (Mielke, 2018). The increased life expectancy in females may be responsible for the disproportionate incidence rate, as advanced age significantly increases AD risk (Beam et al., 2018). Thus, the prevention of this pathological accumulation is a highly desirable outcome in a therapeutic AD prevention paradigm, especially in female subjects. The statistically significant reduction of Aβ40 and Aβ42 in females offers promising support for the preventative potential of early intervention with FUS-BBBO in female mice. Although there is no significant difference between the accumulation of Aβ40 and Aβ42 between FUS-BBBO and control male mice, this lack of difference is likely attributable to the lack of significant pathological accumulation overall in 6-month-old male mice (Mastrangelo and Bowers, 2008). Female control 3xTg mice had significantly greater concentrations of Aβ40 (*P* = 0.005, unpaired *t*-test) and Aβ42 (*P* = 0.0002, unpaired *t*-test) than control male 3xTg mice, demonstrating the increased disease progression in female compared to male 3xTg mice (Figures 6A–C). A lack of pathological accumulation and disease progression in males narrows the window of preventative therapeutic potential, justifying the results presented herein which demonstrate significant cognitive and pathological benefits in 3xTg females, and limited benefits in 3xTg males. These data indicate that 6 months of age might be too young to observe preventative therapeutic benefit in 3xTg males, which have reduced pathology at that age compared to females in the 3xTg mouse model (Clinton et al., 2007; Mastrangelo and Bowers, 2008; Javonillo et al., 2022).

The reduction in tau in both female and male FUS-BBBO mice demonstrates another promising trend where treatment with FUS-BBBO alone, beginning prior to disease symptom onset, is capable of preventing pathological accumulation. In terms of the progression of tau accumulation in female compared to male 3xTg mice, there is no significant difference between control mice of each gender (Figure 6C). This suggests that total tau accumulation may be developed comparably in 6-month-old male and female mice. However, the lack of significant difference between FUS-BBBO and control for both male and female mice suggests that perhaps FUS-BBBO is less effective at preventing the accumulation of axonal tau than it is of Aβ. Still, the compelling decreasing trend for both male and female indicates that there is potential for some preventative benefit in both.

The FUS and AD fields have historically found common interest in treating progressed AD patients in the clinic. However, increasing evidence about the importance of preserving neuronal pathways prior to neurodegeneration and the uncertainty about the effectiveness of targeting hallmark AD pathologies exclusively has motivated the search for an early intervention for AD. In this study we present FUS as a potential answer to this call. With demonstrated neuropsychiatric, cognitive, and pathological benefits, early intervention with FUS offers promise for clinical translation to populations known to be susceptible to AD.

## 5. Conclusion

In conclusion, the results presented herein demonstrate that FUS-BBBO is capable of affecting early mechanisms in AD progression to prevent the anxieties, cognitive decline, and pathological accumulation that would otherwise present in AD mice. These results offer a promising direction for non-invasive preventative therapy for AD patients, especially those who are at high risk for disease progression. The ideal patient population for this kind of intervention may include aged patients, given that age is the most dominant predictive factor for the development of AD, or most likely, patients who carry one or multiple of the identified AD-associated risk genes. This study may further serve as motivation to explore the utility of FUS as a non-invasive preventative intervention for other neurodegenerative diseases such as Parkinson’s disease or Amyotrophic Lateral Sclerosis. One key limitation of this study was the insignificant memory effects of FUS-BBBO in male mice. This lack of significance may be a consequence of the delayed disease progression in the male 3xTg model at the early age investigated here. Further iterations should be performed in male mice either beginning at a later age or extending beyond the 5 months of treatment administered here to characterize the protective effects of FUS in this population more completely. This study was designed to harness well-characterized positive effects of FUS such as neurogenesis and neuroimmune activation, however, future work includes measuring and correlating the extent of neuroprotection resulting from FUS with individual behavioral performance. Future work will also include further characterization of the optimal treatment paradigm and timing regimen for preventative intervention with FUS at a human-scaled rate of disease progression. Ongoing clinical trials evaluating the safety and efficacy of FUS for the treatment of progressed AD have opened the door to further translation of the preventative application presented herein. The continued collaboration of ultrasound scientists with neurologists and the use of patient-specific simulations taking into account individual anatomical differences will enable safe and efficacious adoption of this technique in the clinic. The results of the present study offer preliminary evidence for the utility of FUS-BBBO as a preventative therapeutic for both cognitive and pathological hallmarks of AD.

## Data availability statement

The raw data supporting the conclusions of this article will be made available by the authors, without undue reservation.

## Ethics statement

This animal study was reviewed and approved by the Institutional Animal Care and Use Committee, Columbia University.

## Author contributions

EK and RN performed the study conceptualization and wrote the text. RN performed the FUS-BBBO, protein quantification, immunohistological staining, imaging, and behavioral analysis. RN and AB performed the T1-weighted MRI acquisition. RN and SG conducted the behavioral experiments. AB and SG performed the animal perfusions. All authors contributed, reviewed, and approved the submitted article.

## Footnotes

1
https://www.who.int/news-room/fact-sheets/detail/dementia
