# Supplementary material for: Getting ahead of Alzheimer’s disease: early intervention with focused ultrasound
Source: Front Neurosci. 2023 Jul 27;17:1229683. doi: 10.3389/fnins.2023.1229683 (PMC10412991; doi:10.3389/fnins.2023.1229683)
Supplement: Supplementary file 1 [file Data_Sheet_1.docx]

Supplementary Material

Early intervention with focused ultrasound-induced blood-brain barrier disruption slows cognitive impairment and Alzheimer’s Disease progression in 3xTg mice

**Rebecca L. Noel^1^*, Samantha L. Gorman^1^, Alec J. Batts^1^ & Elisa E. Konofagou^1,2^***

*** Correspondence:**

Rebecca Noel: rln2119@columbia.edu

Elisa E. Konofagou: ek2191@columbia.edu

# Supplementary Figures

**
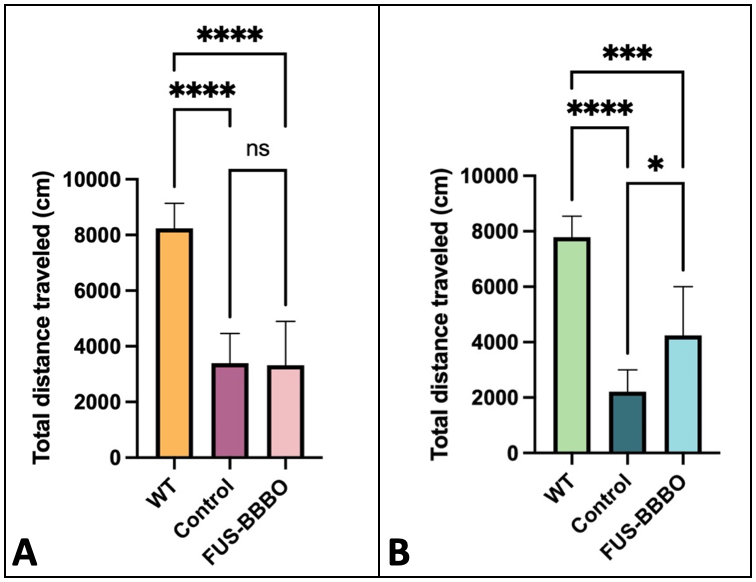
**

**Figure S1:** Distance traveled in open field test. (A-B) Average distance traveled by mice in each cohort are shown for females (A) and males (B) over the 20-minute Open Field test. Statistically significant differences are determined by one-way ANOVA with multiple comparisons.

**
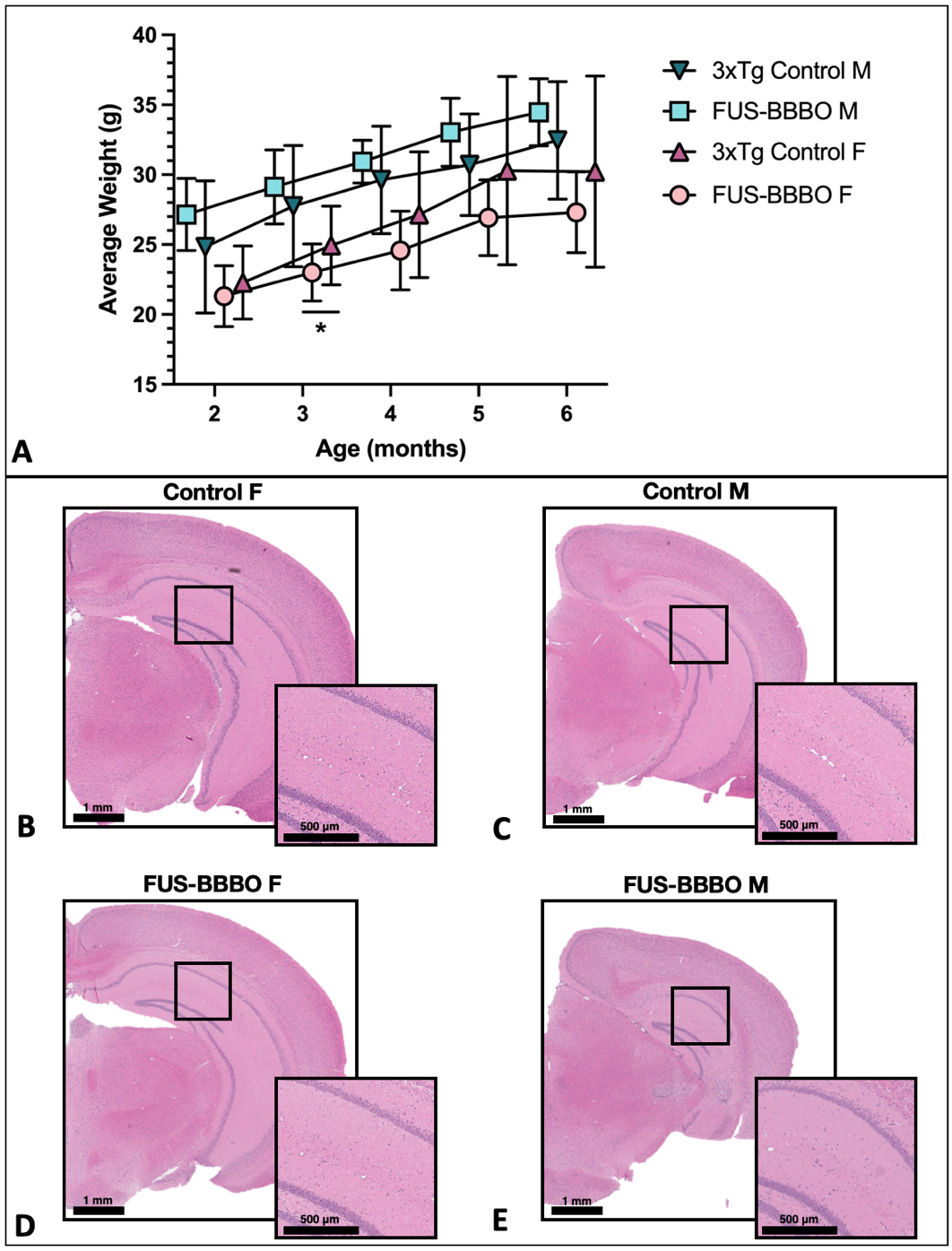
**

**Figure S2: Repeated FUS-BBBO does not negatively affect animal health.** (A) The weights of all 3xTg subjects were taken monthly as a measure of health throughout the five months of sonications. No significant difference was found between age- and gender-matched cohorts besides at 3 months of age where the control females weighed more than FUS-BBBO females (unpaired t test). H&E images reveal no red blood cell extravasation or damage in any of the 3xTg cohorts (B-E).

**
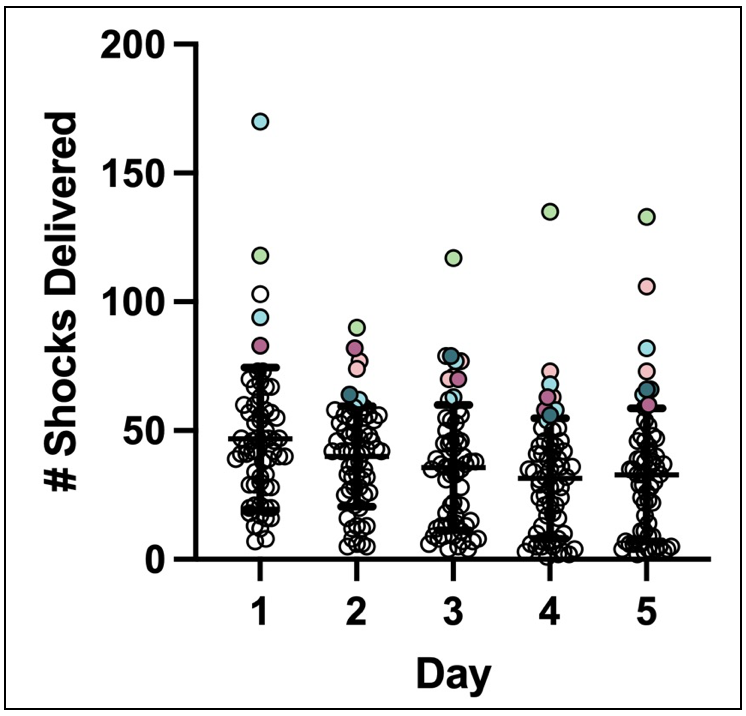
**

**Figure S3: Behavior exclusion criteria.** All mice were combined together. Mice who received more shocks than 1 standard deviation above the group mean for ≥ 2 out of 5 training days were omitted for their inability to learn the paradigm. The data points that qualified these mice for omission are shaded according to their experimental group.

**
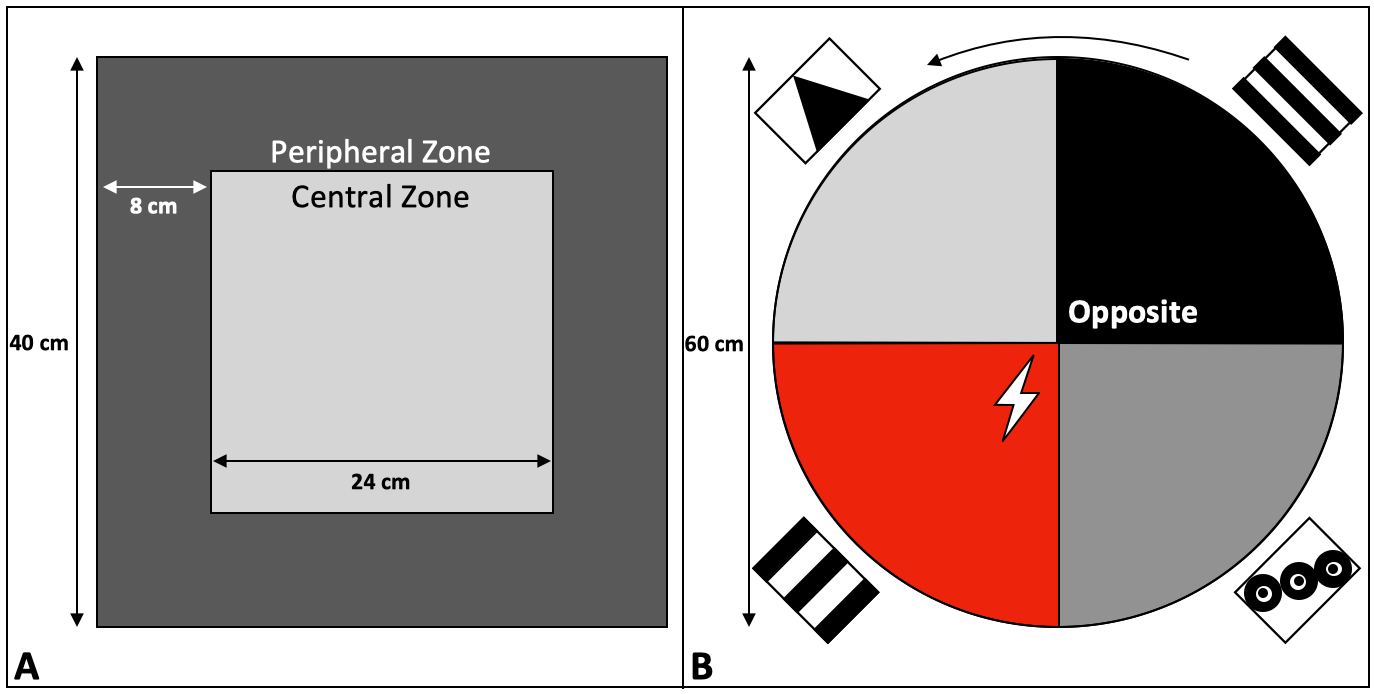
**

**Figure S4: Behavioral apparatus specifications.** (A) Open field test dimensions. (B) Active place avoidance dimensions.

**
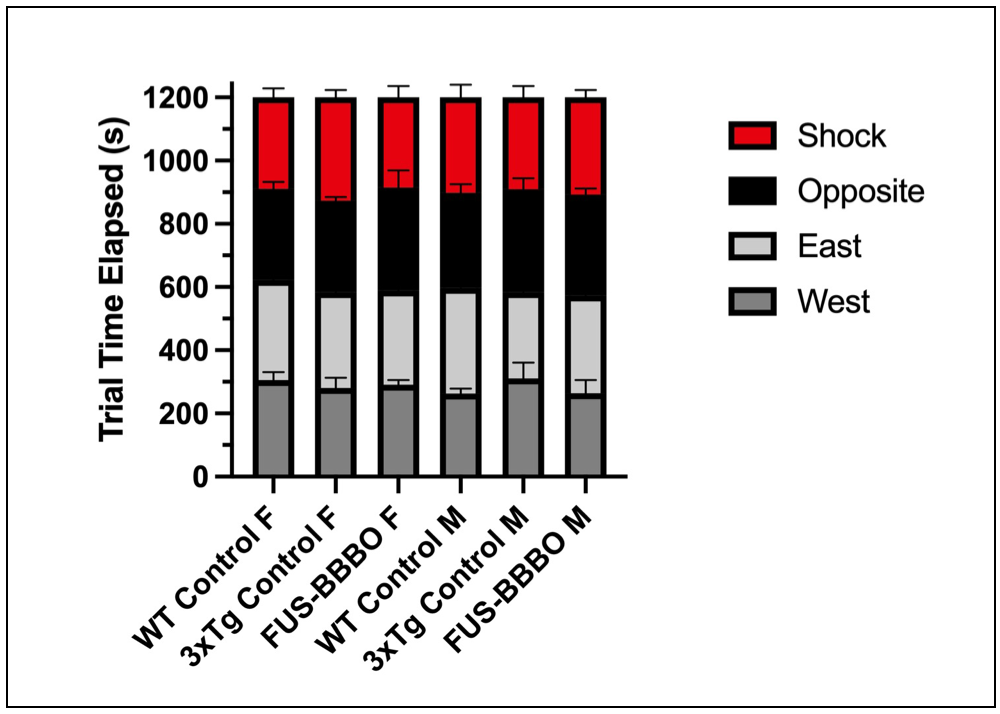
**

**Figure S5: APA Habituation time per quadrant reveals no arena bias.** The time elapsed in each quadrant of the APA arena during the habituation trial for each cohort is shown. Error bars indicate group standard deviation.
